# Estimating the Proportion of COVID-19 Contacts Among Households Based on Individuals With Myocardial Infarction History: Cross-sectional Telephone Survey

**DOI:** 10.2196/26955

**Published:** 2021-04-27

**Authors:** Laurie Fraticelli, Julie Freyssenge, Clément Claustre, Mikaël Martinez, Abdesslam Redjaline, Patrice Serre, Thomas Bochaton, Carlos El Khoury

**Affiliations:** 1 RESCUe-RESUVal Lucien Hussel Hospital Vienne France; 2 Laboratory Systemic Health Care P2S EA 4129 University of Lyon Lyon France; 3 RESHAPE Research on Healthcare Performance INSERM U1290 Université Claude Bernard Lyon 1 Lyon France; 4 Emergency Department Hospital Center du Forez Montbrison France; 5 REULIAN Emergency network Loire Ardèche Nord Firminy France; 6 Emergency Department Hospital Center Le Corbusier Firminy France; 7 Emergency Department Hospital Center Fleyriat Bourg-en-Bresse France; 8 Emergency and Critical Cardiac Care Cardiologic Hospital Hospices Civils de Lyon Bron France; 9 Emergency Department and Clinical Research Unit Medipole Hopital Mutualiste Villeurbanne France

**Keywords:** COVID-19, survey, myocardial infarction, cases, contacts, household, estimate, cross-sectional, cardiovascular, risk, symptom

## Abstract

**Background:**

Adults with cardiovascular diseases were disproportionately associated with an increased risk of a severe form of COVID-19 and all-cause mortality.

**Objective:**

The aims of this study are to report the associated symptoms for COVID-19 cases, to estimate the proportion of contacts, and to describe the clinical signs and behaviors among individuals with and without myocardial infarction history among cases and contacts.

**Methods:**

A 2-week cross-sectional telephone survey was conducted during the first lockdown period in France, from May 4 to 15, 2020. A total of 668 households participated, representing 703 individuals with pre-existing cardiovascular disease in the past 2 years and 849 individuals without myocardial infarction history.

**Results:**

High rates of compliance with health measures were self-reported, regardless of age or risk factors. There were 4 confirmed COVID-19 cases that were registered from 4 different households. Based on deductive assumptions of the 1552 individuals, 9.73% (n=151) were identified as contacts, of whom 71.52% (108/151) were asymptomatic. Among individuals with a myocardial infarction history, 2 were COVID-19 cases, and the estimated proportion of contacts was 8.68% (61/703), of whom 68.85% (42/61) were asymptomatic. The cases and contacts presented different symptoms, with more respiratory signs in those with a myocardial infarction history.

**Conclusions:**

The telephone survey could be a relevant tool for reporting the number of contacts during a limited period and in a limited territory based on the presence of associated symptoms and COVID-19 cases in the households. This study advanced our knowledge to better prepare for future crises.

## Introduction

With more than 100 million confirmed cases worldwide, COVID-19 has caused more than two million deaths in the world from December 2019 to May 2020 [1]. With the appearance of the first cases on January 24, 2020, the health situation in France rapidly deteriorated as in most neighboring countries. The cumulative incidence rate exceeding 10 COVID-19 cases per 100,000 inhabitants in some areas during the week of March 10, 2020 [2]. The French government announced a strict lockdown period from March 17 to May 11, 2020 (ie, 1 month and 25 days) [3].

The need for early and accurate diagnosis for suspected cases become obvious for effective management and for keeping control of the disease spread [4]. Virological tests (reverse transcription polymerase chain reaction [RT-PCR]) have routinely been used to confirm diagnosis, providing results within a few hours. Recent studies have revealed that the computed tomography (CT) scan of the chest was more sensitive but did not replace the RT-PCR that remained the gold standard (sensitivity about 60%-71%) in diagnosing patients with a COVID-19 infection [5,6]. Although serological tests can inform if individuals were exposed to the virus and if they presumably developed immunity, the poor analytical performance can create confusion and may lead to false reassurances, especially when carried out on large populations that have yet to be exposed to the virus and in the absence of a gold standard comparative method [7] at the time of this study. When a COVID-19 diagnosis has been confirmed, chest CT has a central place in the management of respiratory symptoms but cannot be generalized at the scale of the whole population. Therefore, the need to find a more reliable method for estimating prevalence had to be addressed.

At the time of this study, the potential of population surveys in identifying COVID-19 contacts among households may have been underestimated. Existing surveys were designed to assess qualitative data such as risk perception, social isolation, or behavioral disorders [8,9]. Since the symptoms are now well documented in the literature [10] and the tests are more widespread, telephone surveys could also be useful to estimate symptomatic and asymptomatic COVID-19 contacts. Moreover, the most severe forms of COVID-19 and the overall risk of all-cause mortality were disproportionately associated with older adults because of age and pre-existing conditions [11-14].

The main objective was to report the diagnosed COVID-19 cases and the associated symptoms in households with at least one individual with pre-existing myocardial infarction. The second objectives were to estimate the symptomatic and asymptomatic contacts during the 55-day lockdown period in France (March 17 to May 11, 2020) based on deductive assumptions and to describe the clinical signs and behaviors among individuals with or without myocardial infarction history among the cases and contacts.

## Methods

### Study Design

A 2-week cross-sectional telephone survey was conducted, from May 4 to 15, 2020, corresponding to the last week of lockdown in France and the 5 following days (the average period of incubation [15]). The sample comprised households with at least one individual with pre-existing myocardial infarction, collected in the *Observatoire des Syndromes Coronariens Aigus du Réseau Cardiologie Urgence (OSCAR, RESCUe)* (OSCAR) registry and occurring in the Auvergne Rhône-Alpes region in France.

### Sample Selection

The eligible households were identified through inclusions in the OSCAR registry, a multicentric prospective observational registry of the regional emergency cardiovascular network (RESCUe) [16]. Funded by the Regional Agency for Health (*Agence Régionale de Santé Auvergne Rhône-Alpes*), the network covers 3 million inhabitants located in the second most important region in France, including 10 large volume hospitals, representing more than 400 percutaneous coronary interventions per year. The OSCAR registry was approved by the French National Commission of Informatics and Liberties (Commission Nationale de l’Informatique et des Libertés; number 2013090 v0), and all the participants gave informed and oral consent. Patients included were associated with persistent chest pain with ST segment elevation of at least 2 mm in at least two continuous leads.

In this study, the 1164 myocardial infarctions listed in the OSCAR registry were extracted, which occurred between September 3, 2018, and December 10, 2019, discharged at home, and successfully reached by telephone at least once for cardiological follow-up. With events dating less than 2 years and the inclusion criteria chosen, the chances of successfully reaching households by telephone were increased. The patients discharged to dependent older adult homes were excluded.

#### Investigator Training

A total of 17 investigators were involved in the telephone interviews. They were trained by a 15-year experienced telephone operator for two 2-hour meetings with practical instructions (see Multimedia Appendix 1) and simulations by role playing exercises. The training sessions included information on the context to setting up the survey, the methodology, the construction of the sampling frame and eligibility criteria, the conduct of the questionnaire, and the contact phase. After a brief presentation of the study, investigators collected the oral consent from the first respondent to allow the collection of anonymous data for all individuals living in the household during the lockdown period. Interviews took place between 10 AM and noon, and between 1 PM and 6 PM. If no response was received, three other telephone attempts were made at different time slots and days.

#### Ethical Approval

In accordance with French regulations, an individual information note was addressed, after the telephone interview, by email or postal mail to the household to explain the purpose of the study and the rights after data collection.

#### Data Collection

The survey items were elaborated based on a scoping review of the PubMed scientific literature and depending on the World Health Organization symptom list, as updated at the time of the study. The item selection was validated by two emergency physicians, especially the symptoms and the disease’s history (see Multimedia Appendix 1). The investigator collected information from the first individual of the household who picked up the phone. This first respondent answered for all the individuals living in the household. The questionnaire comprised of a common part relating to the household in general and another part relating to each individual living in the household during the lockdown period. The common part relating to the household consisted of identifying the place of residence (zip code and city name), the number of individuals, and any possible regular contacts of a third person (home nurse or home helper). In addition to the items on COVID-19 symptoms observed since March 1, 2020, the questionnaire for individuals comprised sociodemographic items (age, sex, weight, height, occupation), the respect of precautionary behaviors (physical distancing, contact outside home, number of outings per week), pre-existing comorbidities and treatments, travels to high risk areas in France or abroad, and the results of nasal or blood testing or chest CT scan. Moreover, it included the delivered, reported, or renounced consultation during the lockdown period and if another individual of the household was hospitalized or deceased from COVID-19.

#### Definition and Assumptions

When an individual was confirmed to be COVID-19 positive, all the other individuals living in the household were considered as contacts [17,18]. A COVID-19 contact was also defined when they had been in contact with a confirmed case since March 1, 2020, or had a relative from the same household not present at the time of the survey who was hospitalized or deceased from COVID-19.

#### Statistical and Geographical Analysis

To determine the representativeness of the study sample, the open-source data from the French Institute of Statistics and Economic Studies was used to compare the respondents included in the survey to the inhabitants living in the same area, based on a two-stage approach by age [11] and sex [19].

To report the proportion of COVID-19 cases and to estimate the contacts, baseline characteristics in numbers and percentages were provided for categorical variables and medians and IQRs for continuous variables. Bivariate analyses were assessed using the Fisher exact test for categorical variables and the nonparametric Wilcoxon rank test for continuous variables. Statistical analyses were performed using R 3.6.2 software (R Foundation for Statistical Computing). The level of significance was set at a *P* value <.05. When frequencies were insufficient (<10) to provide a statistical test value, an em dash was used.

The missing data represented less than 1%, except for the BMI (10.12%). The denominator was specified when different from the total number.

To describe the clinical signs among symptomatic individuals, a network-based approach [20] was used where the nodes represented an association of symptoms (reported by at least two individuals), linked by shared symptoms. Individuals with and without myocardial infarction history were compared. As the small number of observations did not allow for the proposal of a statistical test, a descriptive approach was proposed to represent the common symptoms.

## Results

### Inclusions

**Figure 1:**
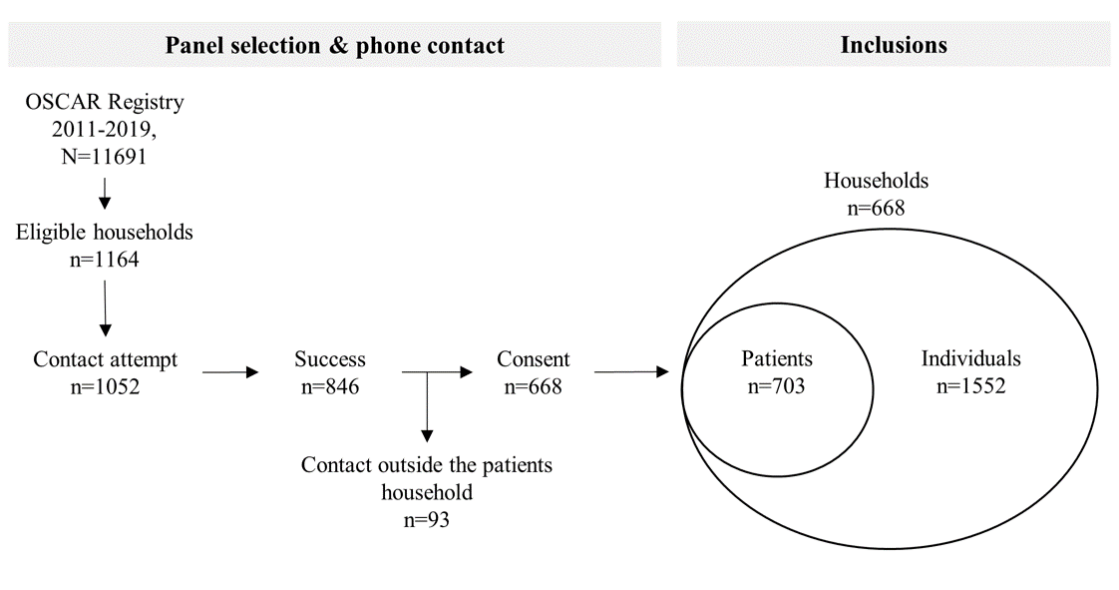
Flowchart of the panel sample selection and the final inclusions households and individuals with myocardial infarction history (patients) and without myocardial infarction history. OSCAR: Observatoire des Syndromes Coronariens Aigus du Réseau Regional Emergency Cardiovascular Network.

A total of 1164 eligible households from the OSCAR registry were identified. The investigators made 1052 call attempts in a 2-week telephone survey, with an average of 1.63 calls per household. A total of 668 households gave their consent to participate, representing 1552 individuals (Figure 1).

**Figure 2:**
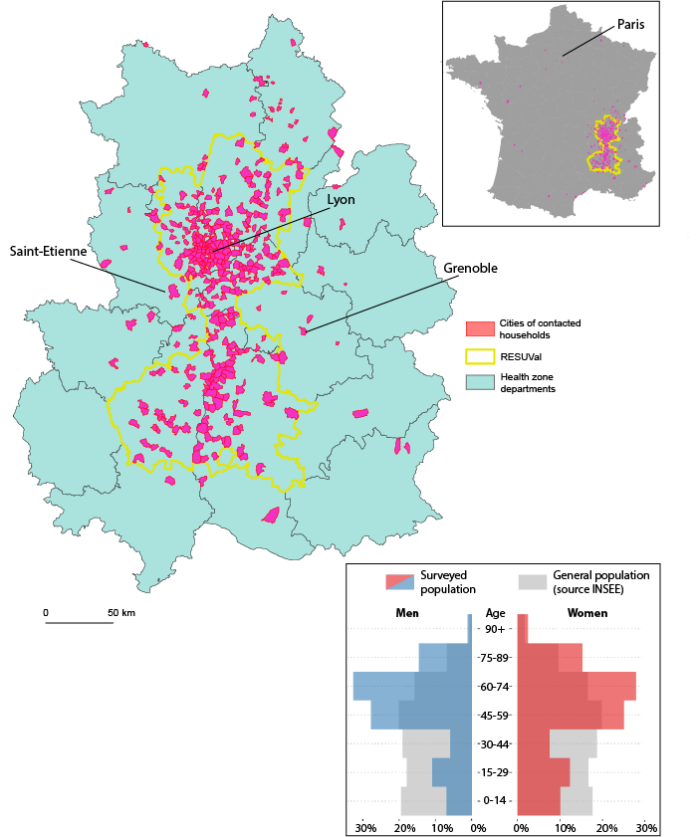
Territorial coverage and age and sex representativeness of the households included in the telephone survey (N=1552 in France for n=1507 in the RESUVal area and its adjacent departments). INSEE: Institut national de la statistique et des études économiques.

The initial response rate was about 63.49% (668/1052) and the participation rate was about 88.70% (668/753). A total of 134 individuals living alone during the lockdown period (ie, 20.06% of the 668 households) was observed. Additionally, 703 individuals with a myocardial infarction history were reported (ie, 45.30% of the 1552 individuals). The proportions of women (777/1552, 50.06%) and men (775/1552, 49.94%) were balanced. However, the study sample included older individuals compared to the resident households of the area (Figure 2).

The sample of men aged 30-44 years was three times less than the inhabitants of the survey area, and there were twice as many men between the ages of 60-64 years.

### Risk Behavior and Risk Factors

High rates of compliance with health measures were self-reported by individuals, regardless of age or risk factors. Among the 1373 individuals who did not work at their usual workplace (ie, 88.47% of the total 1552 sample), 98.69% (n=1355) were confined, 98.69% (n=1355) maintained physical distances, 29.42% (n=404) had no contact with individuals outside their household during the lockdown period, and 23.89% (n=328) went out only once per week. Only 14.2% (n=95) of the 668 households were regularly visited by a caregiver or a nurse. Among the individuals at risk (age ≥60 years with at least one comorbidity; n=709), 98.59% (n=699) complied with the lockdown and 99.15% (n=703) complied with keeping physical distances.

Less than a third of the 1552 individuals were vaccinated against influenza A H1N1 (n=510, 32.86%). This proportion reached 49.93% (n=351) among the 703 individuals with myocardial infarction history. Nearly a quarter (n=314, 20.23%) of the sample presented at least one COVID-19 symptom. There were more symptomatic individuals with myocardial infarction history observed (169/703, 24.04% vs 143/847, 16.88%; *P*<.001). These groups of individuals were significantly associated with different risk factors and comorbidities (Table 1).

**Table 1:** Risk factors and history of the 1552 included individuals with and without myocardial infarction history (two missing values).

Characteristics		Without myocardial infarction history		With myocardial infarction history		*P* value for total	*P* value for symptomatic
		Total (n=847)	Symptomatic (n=143)	Total (n=703)	Symptomatic (n=169)		
**Risk factors**							
	Age (years), median (IQR)	49 (19-65)	56 (43-69)	64 (55-74)	63 (55-74)	<.001	<.001
	Active smoker (age≥15 years), n/N (%)	138/716 (19.27)	23/137 (16.79)	83/701 (11.84)	24/168 (14.29)	<.001	.63
	BMI≥30, n/N (%)	73/733 (9.96)	18/136 (13.24)	92/661 (13.92)	26/162 (16.05)	.02	.51
	Hypertension, n (%)	98 (11.57)	22 (15.38)	176 (25.04)	54 (31.95)	<.001	<.001
	Diabetes, n (%)	53 (6.26)	12 (8.9)	118 (16.79)	35 (20.71)	<.001	<.001
	Heart failure, n (%)	6 (0.71)	4 (2.80)	64 (9.10)	30 (17.75)	—^a^	—
**History, n (%)**							
	Asthma	23 (2.72)	6 (4.20)	17 (2.42)	6 (3.55)	.75	—
	Rheumatism/polyarthritis	26 (3.07)	13 (9.09)	35 (4.98)	22 (13.02)	.75	.77
	Cancer	23 (2.72)	6 (4.20)	33 (4.69)	8 (4.73)	.04	—
	Hypothyroidism	30 (3.54)	13 (9.09)	16 (2.28)	6 (3.55)	.17	—
	Stroke/stroke-like	6 (0.71)	3 (2.10)	32 (4.55)	5 (2.96)	—	—
	Renal disease	4 (0.47)	2 (1.40)	21 (2.99)	7 (4.14)	—	—
	Respiratory failure	7 (0.83)	0 (0.00)	18 (2.56)	8 (4.73)	—	—
	Neurologic disease	8 (0.94)	1 (0.70)	13 (1.85)	7 (4.14)	—	—
	Chronic obstructive pulmonary disease	5 (0.59)	2 (1.40)	11 (1.56)	4 (2.37)	—	—
	Immune disease	5 (0.59)	1 (0.70)	7 (1.00)	2 (1.18)	—	—
	Liver disease	3 (0.35)	1 (0.70)	5 (0.71)	0 (0.00)	—	—
	Emphysema	2 (0.24)	1 (0.70)	5 (0.71)	1 (0.59)	—	—
	Oxygen at home	1 (0.12)	0 (0.00)	4 (0.57)	1 (0.59)	—	—
	Other (ie, dyslipidaemia)	39 (4.60)	12 (8.39)	57 (8.11)	14 (8.28)	<.001	>.99

^a^Not available because frequencies were insufficient to provide a statistical test value.

### Symptomatology

The proportion of individuals associated with COVID-19–like symptoms was 20.23% (314/1552), of whom 50.3% (158/314) reported only one symptom. Among the 314 symptomatic individuals, the most frequent symptoms were cough (n=93, 29.6%), headaches (n=85, 27.1%), runny nose (n=79, 25.2%), unusual fatigue (n=65, 20.7%), fever (n=58, 18.5%), and sore throat (n=49, 15.6%).

**Figure 3:**
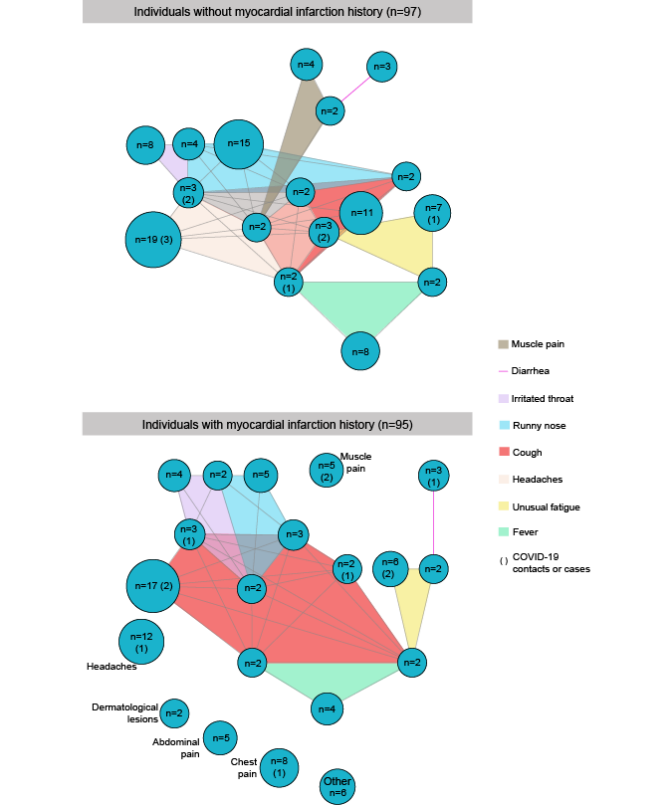
Symptom network of associations reported by at least two individuals: comparison of the reported clinical signs between individuals without myocardial infarction history (n=97) and with myocardial infarction history (n=95).

A total of 37 distinct symptom associations were reported by at least two individuals (Figure 3), 20 associations for individuals with myocardial infarction history (n=95), and 17 associations for individuals without myocardial infarction history (n=97).

Individuals with myocardial infarction history were more associated with an isolated symptom compared to other individuals, accounting for 40% (38/95): headache, chest pain, muscle pain, abdominal pain, and dermatological lesions. To illustrate, among individuals without pre-existing myocardial infarction, headaches were frequently associated with other symptoms like runny nose, cough, sore throat, fever, unusual fatigue, or muscle pain. Among individuals with myocardial infarction history, the most associated symptom was cough with sore throat, runny nose, unusual fatigue, and fever. Among the 47 symptomatic individuals (43 contacts and 4 cases), 20 were observed at least twice (in parenthesis in Figure 3).

### Consultation and Diagnostic Tests

**Figure 4:**
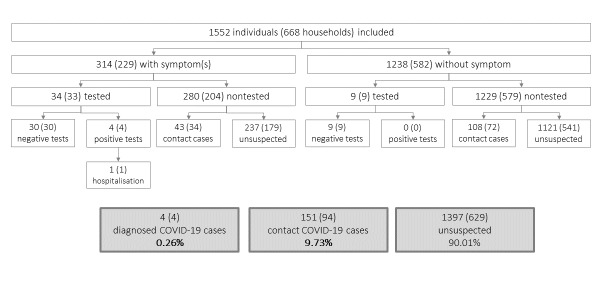
Final reporting of the testing process among individuals and households sampled.

Only 38.85% (n=122) of the 314 symptomatic individuals consulted a general practitioner during the lockdown period because of their symptoms; 68.03% (83/122) of them went to the doctor’s office, 15.6% (19/122) used a telemedicine service, and 16.4% (20/122) visited the emergency department. Only 2.77% (43/1552) of individuals were tested. The test processes were RT-PCR in 56% (24/43), chest CT in 23% (10/43), and blood tests in 44% (19/43). Only 34 (10.83%) tests were performed among the 314 symptomatic individuals with four positive results leading to one hospitalization (Figure 4; 1 by RT-PCR, 1 by blood tests, 1 by chest CT scan, and 1 by all three tests).

The proportion of confirmed COVID-19 cases was 0.26% (4/1552) of individuals, and the proportion of contacts was 9.73% (151/1552), of whom 71.52% (108/151) were asymptomatic (Figure 4). In the subgroup of individuals with myocardial infarction history, two individuals were COVID-19 confirmed among four positive tests, 8.68% (61/703) of contacts, of which 68.85% (42/61) were asymptomatic contacts.

### Scheduled Consultations for Patients During the Lockdown Period

Nearly a quarter of individuals (369/1552, 23.78%) had their appointments rescheduled at the physician’s initiative, but these delays were more common among individuals with myocardial infarction history (259/703, 36.8% vs 110/847, 13%; *P*<.001). Among them, 7.54% decided on their own to cancel an appointment (vs 3.78%; *P*=.001), and 9.10% decided to report it (vs 2.72%; *P*<.001). In 76.07% (89/117) of those cases, the appointment was related to a medical follow-up (vs 67.27%; *P*=.25).

## Discussion

### Principal Results

A network-based approach was provided to understand the different clinical signs associated with contacts of COVID-19. Individuals with myocardial infarction history and who were identified as contacts based on deductive assumptions were most likely to have respiratory symptoms such as cough, sore throat, runny nose, and fever, whereas other individuals presented nonspecific signs (eg, headaches or muscle pain).

The initial response rate of 63.49% (668 included households out of 1052 contact attempts) showed that the telephone survey is a suitable and feasible study design to address such a research question in a short period of time [21]. This proportion of participation is probably due to the population availability during the lockdown period and to their high interest in this unprecedented pandemic situation. If a paper questionnaire had been sent by postal envelope or surveyed through an online questionnaire, the participation rate would have been much lower [22]. The period of the survey—the last week of lockdown and first week post lockdown—was considered appropriate to study the lockdown period, as the mean incubation period lasts 5 days [15].

The study sample also provided estimations on adherence to precautionary measures during the lockdown period with a high compliance rate in our high risk population. This compliance with precautionary measures might explain why only one hospitalization was recorded and the absence of death. Relatives that were susceptible had adapted their behavior as to not expose the individual with myocardial infarction history at risk of infection in the household. Nevertheless, it cannot assume that precautions were observed by the whole population, especially among healthier and younger individuals [23].

### Limitations

Although the sample was not representative of the inhabitants of the territory, the included population constitutes an exhaustive population of myocardial infarction history, focusing on middle-aged and older adult individuals. The literature has established that the latter are associated with higher mortality compared to young or middle-aged individuals [23]. The sample study would be associated with higher risk of complications or hospitalization if it included 45.30% of individuals with myocardial infarction history. Previous studies stated that 16.40% of the patients hospitalized with COVID-19 were associated with cardio-cerebrovascular diseases [24]. In addition to these risk factors, of the included individuals, 15.60% were active smokers, 11.84% were obese, and 11.03% had diabetes [25-27].

In addition, the public health measures that aim to prevent or control transmission in the community [28] were hammered at the same time by the mainstream media and by health professionals [29], and were compulsory under penalty in public places in France. As a consequence, there might be a bias since respondents may have felt compelled to self-report that they respected them [30].

### Comparison With Prior Work

Since the prevalence of COVID-19 is unknown, this cross-sectional survey was considered to be a useful and rapid means of understanding the pandemic situation at a given time and local place [31]. In France, a telephone survey was conducted in an emergency medical dispatching center at the same period of this study, based on COVID-19 cases only, to better characterize the patients managed in an outpatient setting 12 hours after positive testing [21]. Considered as a telemedicine solution, the telephone survey is an alternative to face-to-face consultations during the COVID-19 pandemic [9], and self-reported symptoms are nonetheless clinical signs possibly associated with COVID-19 [32].

Although time series models allow us to understand the trends of the outbreak and to estimate the different epidemiological stages, there is still a need of a reliable and easy to implement solution to evaluate the situation in a high risk population. These findings were not intended to be extrapolated in a predictive model given that the epidemic was spreading unevenly throughout France. Conducted at the end of the lockdown period in France, this survey was presumed to be relevant for a limited area due to the uneven distribution of infection rates across France. Indeed, the Auvergne Rhône-Alpes region was moderately affected compared to the Grand Est and Ile de France regions in the same period [33]. Nevertheless, outcomes such as risk behaviors and symptoms remain declarative responses and subject to approximations.

### Conclusions

A cross-sectional telephone survey was conducted by selecting households from a prospective observational registry of individuals with myocardial infarction history. A low proportion of COVID-19 diagnosis tests was observed, with only 10.62% (34/320) of symptomatic individuals who had been tested over the study period. The estimated proportion of contacts was about 8.68% (61/703) of the respondents with prior history of myocardial infarction, of which 68.85% (42/61) were asymptomatic. These estimates were relevant at several levels; first, they showed that a telephone survey could be a relevant tool for rapidly assessing the number of contacts on a limited territory; second, they could be a useful tool for the local institutional structures for advising and reporting the current situation. In addition to keeping a social link during lockdown and with high risk populations in our territory, these estimates advanced our knowledge to better prepare for future crises.

## Appendix

Multimedia Appendix 1 Phone survey.

## Abbreviations

CT: computed tomography
OSCAR: Observatoire des Syndromes Coronariens Aigus de RESCUe
RESCUe: Réseau Cardiologie Urgence / Regional emergency cardiovascular network
RT-PCR: reverse transcription polymerase chain reaction

## References

[1] (2021). WHO Coronavirus (COVID-19) Dashboard. World Health Organization.

[2] (2021). COVID-19 Point épidémiologique - Situation au 10 mars 2020 à minuit. Santé publique France.

[3] (2021). Point épidémio régional Auvergne-Rhône-Alpes Spécial COVID-19 7 mai 2020. Santé publique France.

[4] Ceylan Z (2020). Estimation of COVID-19 prevalence in Italy, Spain, and France. Sci Total Environ.

[5] Fang Y, Zhang H, Xie J, Lin M, Ying L, Pang P, Ji W (2020). Sensitivity of chest CT for COVID-19: comparison to RT-PCR. Radiology.

[6] Miller TE, Garcia Beltran WF, Bard AZ, Gogakos T, Anahtar MN, Astudillo MG, Yang D, Thierauf J, Fisch AS, Mahowald GK, Fitzpatrick MJ, Nardi V, Feldman J, Hauser BM, Caradonna TM, Marble HD, Ritterhouse LL, Turbett SE, Batten J, Georgantas NZ, Alter G, Schmidt AG, Harris JB, Gelfand JA, Poznansky MC, Bernstein BE, Louis DN, Dighe A, Charles RC, Ryan ET, Branda JA, Pierce VM, Murali MR, Iafrate AJ, Rosenberg ES, Lennerz JK (2020). Clinical sensitivity and interpretation of PCR and serological COVID-19 diagnostics for patients presenting to the hospital. FASEB J.

[7] Diamandis P, Prassas I, Diamandis EP (2020). Antibody tests for COVID-19: drawing attention to the importance of analytical specificity. Clin Chem Lab Med.

[8] Goodman-Casanova JM, Dura-Perez E, Guzman-Parra J, Cuesta-Vargas A, Mayoral-Cleries F (2020). Telehealth home support during COVID-19 confinement for community-dwelling older adults with mild cognitive impairment or mild dementia: survey study. J Med Internet Res.

[9] Elawady A, Khalil A, Assaf O, Toure S, Cassidy C (2020). Telemedicine during COVID-19: a survey of health care professionals' perceptions. Monaldi Arch Chest Dis.

[10] Ahn D, Shin H, Kim M, Lee S, Kim H, Myoung J, Kim B, Kim S (2020). Current status of epidemiology, diagnosis, therapeutics, and vaccines for novel coronavirus disease 2019 (COVID-19). J Microbiol Biotechnol.

[11] Nanda A, Vura NVRK, Gravenstein S (2020). COVID-19 in older adults. Aging Clin Exp Res.

[12] Aggarwal G, Cheruiyot I, Aggarwal S, Wong J, Lippi G, Lavie CJ, Henry BM, Sanchis-Gomar F (2020). Association of cardiovascular disease with coronavirus disease 2019 (COVID-19) severity: a meta-analysis. Curr Probl Cardiol.

[13] Matsushita K, Ding N, Kou M, Hu X, Chen M, Gao Y, Honda Y, Zhao D, Dowdy D, Mok Y, Ishigami J, Appel LJ (2020). The relationship of COVID-19 severity with cardiovascular disease and its traditional risk factors: a systematic review and meta-analysis. Glob Heart.

[14] Yahia F, Zakhama L, Ben Abdelaziz A (2020). COVID-19 and cardiovascular diseases. Scoping review study. Tunis Med.

[15] Lauer SA, Grantz KH, Bi Q, Jones FK, Zheng Q, Meredith HR, Azman AS, Reich NG, Lessler J (2020). The incubation period of coronavirus disease 2019 (COVID-19) from publicly reported confirmed cases: estimation and application. Ann Intern Med.

[16] El Khoury C, Bochaton T, Flocard E, Serre P, Tomasevic D, Mewton N, Bonnefoy-Cudraz E, Observatoire des Syndromes Coronaires Aigus dans RESCUe (OSCAR) Research Team (2017). Five-year evolution of reperfusion strategies and early mortality in patients with ST-segment elevation myocardial infarction in France. Eur Heart J Acute Cardiovasc Care.

[17] Zhang J, Tian S, Lou J, Chen Y (2020). Familial cluster of COVID-19 infection from an asymptomatic. Crit Care.

[18] Takyi-Williams J (2020). Household representative sample strategy for COVID-19 large-scale population screening. Med Hypotheses.

[19] Elgendy IY, Pepine CJ (2020). Why are women better protected from COVID-19: clues for men? Sex and COVID-19. Int J Cardiol.

[20] Faisandier L, Bonneterre V, De Gaudemaris R, Bicout DJ (2011). Occupational exposome: a network-based approach for characterizing occupational health problems. J Biomed Inform.

[21] Lapostolle F, Schneider E, Vianu I, Dollet G, Roche B, Berdah J, Michel J, Goix L, Chanzy E, Petrovic T, Adnet F (2020). Clinical features of 1487 COVID-19 patients with outpatient management in the Greater Paris: the COVID-call study. Intern Emerg Med.

[22] Campbell N, Ali F, Finlay AY, Salek SS (2015). Equivalence of electronic and paper-based patient-reported outcome measures. Qual Life Res.

[23] Liu K, Chen Y, Lin R, Han K (2020). Clinical features of COVID-19 in elderly patients: a comparison with young and middle-aged patients. J Infect.

[24] Li B, Yang J, Zhao F, Zhi L, Wang X, Liu L, Bi Z, Zhao Y (2020). Prevalence and impact of cardiovascular metabolic diseases on COVID-19 in China. Clin Res Cardiol.

[25] Engin AB, Engin ED, Engin A (2020). Two important controversial risk factors in SARS-CoV-2 infection: obesity and smoking. Environ Toxicol Pharmacol.

[26] Palaiodimos L, Kokkinidis DG, Li W, Karamanis D, Ognibene J, Arora S, Southern WN, Mantzoros CS (2020). Severe obesity, increasing age and male sex are independently associated with worse in-hospital outcomes, and higher in-hospital mortality, in a cohort of patients with COVID-19 in the Bronx, New York. Metabolism.

[27] Gupta R, Ghosh A, Singh AK, Misra A (2020). Clinical considerations for patients with diabetes in times of COVID-19 epidemic. Diabetes Metab Syndr.

[28] Flaxman S, Mishra S, Gandy A, Unwin HJT, Mellan TA, Coupland H, Whittaker C, Zhu H, Berah T, Eaton JW, Monod M, Ghani AC, Donnelly CA, Riley S, Vollmer MAC, Ferguson NM, Okell LC, Bhatt S, Imperial College COVID-19 Response Team (2020). Estimating the effects of non-pharmaceutical interventions on COVID-19 in Europe. Nature.

[29] Al-Hasan A, Khuntia J, Yim D (2020). Threat, coping, and social distance adherence during COVID-19: cross-continental comparison using an online cross-sectional survey. J Med Internet Res.

[30] Imai N, Gaythorpe KAM, Abbott S, Bhatia S, van Elsland S, Prem K, Liu Y, Ferguson NM (2020). Adoption and impact of non-pharmaceutical interventions for COVID-19. Wellcome Open Res.

[31] Negri E, Scarpino V, La Vecchia C (2021). Prevalence of COVID-19-like symptoms in Italy and Lombardy, March-April 2020, and their implications on cancer prevention, diagnosis and management. Eur J Cancer Prev.

[32] Menni C, Valdes AM, Freidin MB, Sudre CH, Nguyen LH, Drew DA, Ganesh S, Varsavsky T, Cardoso MJ, El-Sayed Moustafa JS, Visconti A, Hysi P, Bowyer RCE, Mangino M, Falchi M, Wolf J, Ourselin S, Chan AT, Steves CJ, Spector TD (2020). Real-time tracking of self-reported symptoms to predict potential COVID-19. Nat Med.

[33] (2021). COVID-19 Point épidémiologique hebdomadaire du 21 mai 2020. Santé publique France.

