# Supplementary material for: Estimating the Proportion of COVID-19 Contacts Among Households Based on Individuals With Myocardial Infarction History: Cross-sectional Telephone Survey
Source: JMIR Form Res. 2021 Apr 27;5(4):e26955. doi: 10.2196/26955 (PMC8083951; doi:10.2196/26955)
Supplement: Multimedia Appendix 1 [file formative_v5i4e26955_app1.docx]

Phone survey - Interview guide

(04/22/2020 version)

Questionnaire date: |__|__| |__|__| 2020

Household number: |__|__|__|__|

Individual number: |__|__|__|__|

*Instructions:*

*- Introduce yourself (last name, first name) on behalf of the regional emergency network,*

*- We are conducting a phone survey to assess the number of persons infected with coronavirus,*

*- Do you agree to answer a few anonymous questions?*

*- We will not collect your last name or your date of birth*

***Questions to the first respondent**

Residence zip code: |__|__|__|__|__| Town of residence: ___________________

Number of persons confined in the household: □1 □2 □3 □4 □5 □6 □7 □8 □9 □10

Is there a regular visit from a home helper / personal care assistant / nurse? □ Yes □ No

***Questionnaire of each individual from the household (the adults respond for the minors)**

Sex: □ Man □ Woman Age: |__|__| years old

Weight:|__|__|__| kg Height:|__|__|__|cm

If woman of childbearing age: Are you pregnant? □ Yes □ No □ Post-partum (< 6 weeks)

If yes: □ T1 □ T2 □T3

Do you comply with lockdown measures? □ Yes □ No

Do you comply with social distancing measures when you go out?  □ Yes □ No

Do you have contact with persons other than those in your household? □ Yes □ No

How many times per week do you go out? □0 □1 □2 □3 □4 □5 □>5

Do you work? □ Yes □ No □ Unemployed □ Retired,

If yes: □ telecommuting □ still work at the usual place

Do you smoke? □ Yes □ No □ Ex-smoker

Are you being treated for any of the following conditions?

🡪 way of the questioning 🡪

□ Heart disease □ Stroke □ Immune disease (HIV)  □ Hypertension

□ Heart failure □ Diabetes □ Kidney disease / Dialysis □ Liver disease

□ Respiratory failure □ On oxygen at home □ Asthma

□ COPD (tobacco-related chronic bronchitis)  □ Emphysema

□ Neurological or neuromuscular disease □ Cancer □ Hypothyroidism

□ Rheumatism or polyarthritis □ Other  □ None

Do you have a long-term treatment with:

□ Cortisone □ Methotrexate □ Ciclosporin

□ Imurel (azathioprine) □ Plaquenil (hydroxychloroquine)

Have you traveled since March 1^st^, 2020? □ Yes □ No

If yes, have you been in any of the following risk areas since March 1^st^, 2020?

□ China □ Singapore □ South Korea □ North of Italy □ Iran

□ Grand Est region (France) □ Department of Oise (France)

Have you had any of the following symptoms at least once since March 15, 2020?

🡪 way of the questioning 🡪

□ Fever □ Headaches □ Cough □ Sore throat □ Runny nose □ Breathing discomfort, shortness of breath □ Loss of sense of smell □ Loss of taste □ Unusual fatigue □ Muscular pain, stiffness □ Chest pain □ Stomach ache  □ Nausea/vomiting □ Diarrhea □ Other □ None

Are you vaccinated against the flu? □ Yes □ No

Coronavirus exposure

Since March 15, 2020:

Have you been in contact with a person with respiratory signs?

□ Yes □ No □ Do not know

Have you been in contact with a person diagnosed with a suspected coronavirus infection?

□ Yes □ No □ Do not know

Have you been in contact with a person with a confirmed coronavirus infection?

□ Yes □ No □ Do not know

In relation to the above symptoms, have you seen a doctor at least once since March 15, 2020? □ Yes □ No

If yes, □ in office? □ in emergency room? □ by telemedicine?

Have you undergone:

A nasopharyngeal swab testing? □ Yes □ No

A chest computed tomography scan? □ Yes □ No

A blood test? □ Yes □ No

Has a diagnosis of coronavirus infection been made? □ Yes □ No

Have you been hospitalized? □ Yes □ No

Have you received any specific treatment?

□ Hydroxychloroquine (Plaquenil) □ Kaletra (lopinavir / ritonavir)

□ Azythromycin (Zythromax)

Has your doctor or the hospital rescheduled a consultation or hospitalization appointment for you since March 1^st^, 2020? □ Yes □ No

Have you ever stopped going to your doctor or emergency room because of lockdown?

□ Yes □ No □ With delay

For what reason? □ follow-up visit □ traumatic emergency □ medical emergency

Has another member of the household not currently present been infected with coronavirus?

□ Yes □ No

Was he/she hospitalized for this reason? □ Yes □ No

Has he/she had any complications? □ Yes □ No

If yes, is he/she deceased? □ Yes □ No

*Instructions:*

*- Thank you for your participation to this phone survey.*
